# Quality Assessment of Ojeok-San, a Traditional Herbal Formula, Using High-Performance Liquid Chromatography Combined with Chemometric Analysis

**DOI:** 10.1155/2015/607252

**Published:** 2015-10-11

**Authors:** Jung-Hoon Kim, Chang-Seob Seo, Seong-Sil Kim, Hyeun-Kyoo Shin

**Affiliations:** ^1^Herbal Medicine Formulation Research Group, Korea Institute of Oriental Medicine, Daejeon 305-811, Republic of Korea; ^2^Division of Pharmacology, School of Korean Medicine, Pusan National University, Yangsan, Gyeongnam 626-870, Republic of Korea

## Abstract

Ojeok-san (OJS) is a traditional herbal formula consisting of 17 herbal medicines that has been used to treat various disorders. In this study, quantitative analytical methods were developed using high-performance liquid chromatography equipped with a photodiode array detector to determine 19 marker compounds in OJS preparations, which was then combined with chemometric analysis. The method developed was validated in terms of its precision and accuracy. The intra- and interday precision of the marker compounds were <3.0% of the relative standard deviation (RSD) and the recovery of the marker compounds was 92.74%–104.16% with RSD values <3.0%. The results of our quantitative analysis show that the quantities of the 19 marker compounds varied between a laboratory water extract and commercial OJS granules. The chemometric analysis used, principal component analysis (PCA) and hierarchical clustering analysis (HCA), also showed that the OJS water extract produced using a laboratory method clearly differed from the commercial OJS granules; therefore, an equalized production process is required for quality control of OJS preparations. Our results suggest that the HPLC analytical methods developed are suitable for the quantification and quality assessment of OJS preparations when combined with chemometric analysis involving PCA and HCA.

## 1. Introduction

Ojeok-san (OJS) is a traditional herbal formula used in Korean medicine that consists of 17 compositional herbal medicines: Atractylodis rhizoma, Ephedrae herba, Citri Unshius pericarpium, Magnoliae cortex, Platycodonis radix, Aurantii Fructus Immaturus, Angelicae gigantis radix, Zingiberis rhizoma, Paeoniae radix, Poria sclerotium, Angelicae dahuricae radix, Cnidii rhizoma, Pinelliae tuber, Cinnamomi cortex, Glycyrrhizae radix et rhizoma,* Zingiberis* rhizoma recens, and Allii fistulosi bulbus. Traditionally, OJS has been used to treat disorders such as fever, anhidrosis, headache, whole body pain, contracture of the nape and neck, vomiting, abdominal and heart pain, and menstrual irregularities [1].

Recent studies have reported on the therapeutic effects of OJS against lumbago and inferior limb pain [2], primary dysmenorrheal [3], clastogenicity [4], and airway inflammation and pulmonary fibrosis [5]. Since a combination of multiple components is considered necessary to exhibit the therapeutic effects of the herbal formula, simultaneous determination of the compositional constituents has been developed for qualitative and quantitative analysis. Several previous studies have analyzed the chemical constituents of OJS using reversed-phase high-performance liquid chromatography (RP-HPLC) coupled with pulsed amperometric detection (PAD) or diode array detection (DAD) [6, 7].

Cluster analysis is a data analysis method used to assign similar objects belonging to the same group and is used in a variety of practical applications like bioinformatics, using chemometric analyses, such as principal component analysis (PCA) and hierarchical clustering analysis (HCA) [8, 9].

PCA is an unsupervised pattern recognition technique and is a useful tool for visualizing similarities or differences in multivariate data [10]. PCA can represent objects or variables on a graph and is used to study the proximity of objects to classify them and to detect atypical objects [11]. HCA is a procedure that has a pyramid-like structure and is a very useful and widely adopted technique in information processing [12]. HCA determines similarities between samples by measuring the distance between all possible sample pairs in a high-dimensional space and any similarities between the samples are represented on two-dimensional diagrams [13]. The HPLC analytical method combined with chemometric analysis has been widely accepted for the quality control of herbal medicines, as it can be part of a powerful strategy to differentiate the source, location, or species in herbal medicines [14–16].

Recently, herbal formulas have been manufactured by pharmaceutical companies in diverse dosage forms, such as powder, granules, and tablets, as these are more convenient and easier to take than traditional decoction forms. However, the compositional herbal ratio or the origin of herbal components of a herbal formula may differ between different companies, and so the formula produced by each company may contain a variety of chemical constituents [17–19]. Such chemical inequalities cannot warrant equivalent therapeutic effects between different herbal formula preparations and may lead to a loss of innate characteristics of a given herbal formula.

Therefore, in this study, we developed analytical methods for the quantification of 19 marker compounds in a laboratory-produced water extract and in commercial granules of OJS using HPLC–PDA. In addition, chemometric analysis data were combined with the quantitative results and employed to assess the quality of OJS preparations via the Pearson correlation coefficient and PCA and HCA data.

## 2. Materials and Methods

### 2.1. Chemicals and Reagents

The HPLC-grade acetonitrile and water used were purchased from JT Baker Inc. (Phillipsburg, NJ, USA) and the guaranteed reagent grade acetic acid used was obtained from Junsei (Chuo-ku, Tokyo, Japan). The gallic acid (**1**), chlorogenic acid (**3**), ferulic acid (**6**), benzoic acid (**8**), neohesperidin (**12**), and cinnamic acid (**15**) used were obtained from Sigma-Aldrich (St. Louis, MO, USA). The protocatechuic acid (**2**) and nodakenin (**9**) used were purchased from ChromaDex (Irvine, CA, USA) and NPC BioTech (Geumsan, Chungnam, Korea), respectively. The albiflorin (**4**), paeoniflorin (**5**), liquiritin (**7**), naringin (**11**), cinnamaldehyde (**17**), and glycyrrhizin (**19**) used were purchased from Wako Pure Chemical Industries (Chuo-ku, Osaka, Japan). The hesperidin (**10**), ononin (**13**), oxypeucedanin hydrate (**14**), byakangelicin (**16**), and benzoylpaeoniflorin (**18**) used were purchased from Chengdu Biopurify Phytochemicals (Chengdu, Sichuan, China).

**Figure 1:**
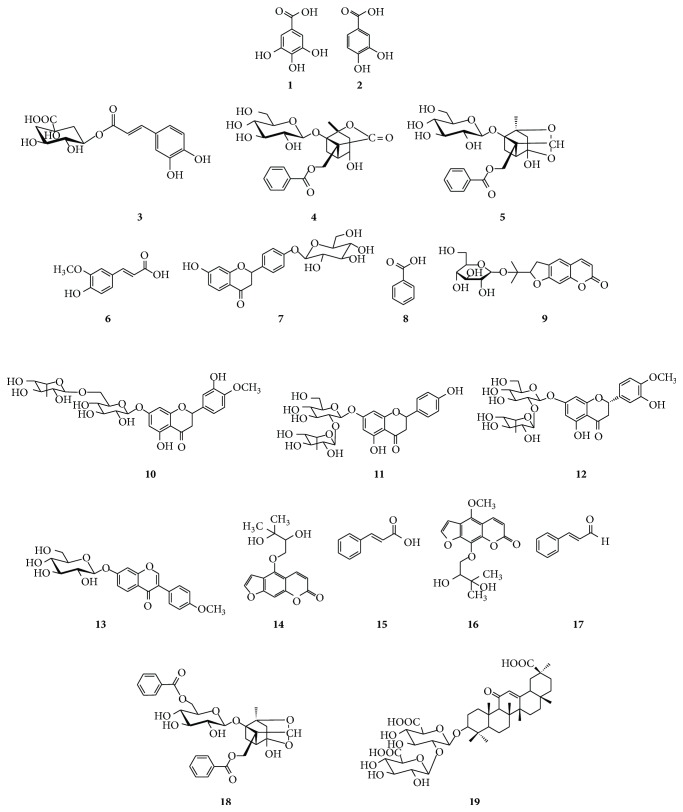
Chemical structures of 19 marker compounds in Ojeok-san (OJS). (**1**) Gallic acid, (**2**) protocatechuic acid, (**3**) chlorogenic acid, (**4**) albiflorin, (**5**) paeoniflorin, (**6**) ferulic acid, (**7**) liquiritin, (**8**) benzoic acid, (**9**) nodakenin, (**10**) hesperidin, (**11**) naringin, (**12**) neohesperidin, (**13**) ononin, (**14**) oxypeucedanin hydrate, (**15**) cinnamic acid, (**16**) byakangelicin, (**17**) cinnamaldehyde, (**18**) benzoylpaeoniflorin, and (**19**) glycyrrhizin.

The purity of the standard compounds was ≥98%; their chemical structures are shown in Figure 1. Compositional herbal medicines of OJS were purchased from the herbal medicine company, Kwangmyungdang Medicinal Herbs (Ulsan, Gyeongbuk, Korea). A voucher specimen (2012-KE04-1–17) was deposited in the Herbal Medicine Formulation Research Group of the Korea Institute of Oriental Medicine. Commercial OJS samples denoted as “OJS02–OJS10” were purchased from nine pharmaceutical companies located in Korea. The compositional herbal ratio was shown in Table 1.

### 2.2. Preparation of the OJS Water Extract and Commercial Granules

Dried herbal drugs consisting of OJS were mixed and extracted using a 10-fold volume of distilled water (w/v) at 100°C for 2 h under pressure (1 kgf/cm^2^) using an electric extractor (COSMOS-660, KyungSeo Machine Co., Incheon, Korea). The extracted decoction was filtered through a standard sieve (number 270, 53 *μ*m, Chunggyesangongsa, Seoul, Korea) and freeze-dried to make OJS water extract powder denoted as “OJS01.”

Powdered OJS01 (200 mg) and commercial OJS granules (OJS02–OJS10, 500 mg) were dissolved in 10 mL of distilled water and the solutions were filtered through a 0.2 *μ*m syringe filter (SmartPor, Woongki Science, Seoul, Korea) before being injected into the HPLC system.

### 2.3. Chromatographic Conditions

The HPLC system used was a Shimadzu LC-20A (Kyoto, Japan) chromatograph equipped with a solvent delivery unit (LC-20AT), an autosampler (SIL-20AC), a column oven (CTO-20A), a degasser (DGU-20A_3_), and a photodiode array detector (SPD-M20A). Separation was conducted on a Gemini C_18_ column (4.6 × 250 mm, 5 *μ*m; Phenomenex, Torrance, CA, USA). The column temperature was set at 40°C. The mobile phase consisted of water containing 0.1% formic acid (A) and acetonitrile (B). The composition of the mobile phase was 6%–20% (B) for 0–20 min, 20%–25% (B) for 25–30 min, 25%–40% (B) for 30–40 min, 40%–46% (B) for 40–50 min, and 46%–87% (B) for 50–55 min, held for 5 min and then reequilibrated to 6% (B) until the end of the analysis. The flow rate was 1.0 mL/min and the injection volume was 10 *μ*L. The detection wavelengths of all standards and samples were in the UV at 230, 250, 260, 270, 275, 280, 290, 310, 325, and 335 nm.

### 2.4. Method Validation

#### 2.4.1. Linearity

The 19 standard compounds were accurately weighed and dissolved in methanol to prepare stock solutions at a concentration of 1000 *μ*g/mL. Stock solutions of the marker compounds were serially diluted to construct calibration curves. The diluted concentrations of marker compounds were plotted against the peak area on the calibration curves and the linearity was measured from the correlation coefficient.

#### 2.4.2. LOD and LOQ

Blank samples were analyzed in triplicate and the area of the noise peak was calculated as the response. The LOD and LOQ were calculated as LOD = 3.3 × SD/*S* and LOQ = 10 × SD/*S*, where SD is the standard deviation of the response and *S* is the slope of the calibration curve.

#### 2.4.3. Precision

The precision was calculated by analyzing sample extracts containing low and high concentrations of the marker compounds. The precision was represented by the relative standard deviation (RSD), which was calculated using the equation RSD = (standard deviation/mean) × 100. The precision was measured three times in a single day (intraday precision) and over three consecutive days (interday precision).

#### 2.4.4. Recovery

The accuracy of the method used was evaluated through the recovery test. Both low and high concentrations of the marker compounds were added to the samples. The recovery was calculated as follows: recovery (%) = ((detected concentration − initial concentration)/spiked concentration) × 100.

### 2.5. Chemometric Analysis

The relationship between OJS samples was evaluated using the Pearson coefficient of the amounts of the marker compounds. To cluster the OJS sample, PCA and HCA were performed based on the rows (OJS samples) and columns (the amounts of the 19 marker compounds). The evaluation of the Pearson coefficient and the clustering analysis (PCA and HCA) were carried out using the open-source software package R (v. 3.0.2).

## 3. Results and Discussion

### 3.1. Optimization of Chromatographic Conditions

The mobile phase modifier, gradient ratio, and UV detection wavelength were considered as the main factors for optimizing the conditions for the HPLC analysis of the OJS water extract. A C_18_ column was employed for the simultaneous determination of the 19 marker compounds in the OJS water extract, as it has been the most frequently used technique in the chemical analysis of herbal medicines [20, 21]. Two different modifiers, 1% acetic acid and 0.1% formic acid, were compared to find the optimal conditions for the separation of the 19 marker compounds. Peak resolution and shape of the marker compounds were considered better indicators when 0.1% formic acid was used as a modifier.

Various ratios of the components of the mobile phase (A : B) were tested using gradient elution, and the optimal separation was observed at the following gradient conditions: 6%–20% (B) for 0–20 min, 20%–25% (B) for 25–30 min, 25%–40% (B) for 30–40 min, 40%–46% (B) for 40–50 min, and 46%–87% (B) for 50–55 min, held for 5 min and then reequilibrated to 6% (B).

**Figure 2:**
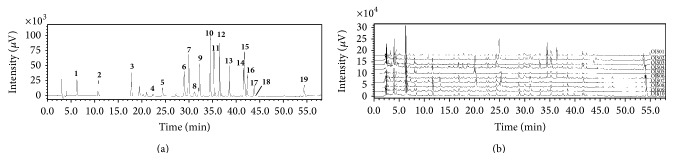
Chromatograms of (a) the standard marker compounds and (b) OJS samples at a detection wavelength of UV 254 nm. (**1**) Gallic acid, (**2**) protocatechuic acid, (**3**) chlorogenic acid, (**4**) albiflorin, (**5**) paeoniflorin, (**6**) ferulic acid, (**7**) liquiritin, (**8**) benzoic acid, (**9**) nodakenin, (**10**) hesperidin, (**11**) naringin, (**12**) neohesperidin, (**13**) ononin, (**14**) oxypeucedanin hydrate, (**15**) cinnamic acid, (**16**) byakangelicin, (**17**) cinnamaldehyde, (**18**) benzoylpaeoniflorin, and (**19**) glycyrrhizin. OJS01, Ojeok-san water extract from the laboratory; OJS02–OJS10, Ojeok-san granules from Korean manufacturers.

The UV wavelength in the range 190–400 nm was scanned to find the maximum absorption for each marker compound. For albiflorin, paeoniflorin, benzoic acid, and benzoylpaeoniflorin, this occurred at 230 nm; for ononin and glycyrrhizin at 250 nm; for protocatechuic acid at 260 nm; for gallic acid and byakangelicin at 270 nm; for liquiritin and cinnamic acid at 275 nm; for hesperidin, naringin, and neohesperidin at 280 nm; for cinnamaldehyde at 290 nm; for oxypeucedanin hydrate at 310 nm; for chlorogenic acid and ferulic acid at 325 nm; and for nodakenin at 335 nm. For the conditions described above, the 19 marker compounds were reasonably separated on C_18_ column for quantitative analysis (Figure 2).

### 3.2. Method Validation

#### 3.2.1. Linear Regression, LOD, and LOQ

The linearity of the calibration curve was measured using the correlation coefficient (*r* ^2^), which ranged in value from 0.9993 to 1.0000 for each compound. The LOD and LOQ values were 0.004–0.090 *μ*g/mL and 0.012–0.272 *μ*g/mL, respectively (Table 2).

#### 3.2.2. Precision and Recovery

The intra- and interday precision, which were represented by the RSD values, were RSD < 3.0% for the two concentration levels (Table 3). The recoveries of the 19 marker compounds were in the range 92.74%–104.16%, with RSD < 4.0% at different spiked concentrations (Table 4). These results indicate that the developed analytical method was precise, accurate, and reliable for the analysis of the 19 marker compounds in the OJS samples.

### 3.3. Quantification of the Marker Compounds in the OJS Samples

The method we established was successfully applied to determine the 19 reference compounds in the OJS water extract (OJS01) and commercial OJS granules (OJS02–OJS10). There was wide variation observed in the contents of the marker compounds in the 10 OJS samples. While OJS01 contained the 19 marker compounds, the commercial OJS granules showed lack of one or more of the following compounds: protocatechuic acid, chlorogenic acid, ferulic acid, nodakenin, hesperidin, neohesperidin, and cinnamaldehyde.

Moreover, variation in the content of these compounds was apparent between the OJS samples: 2.8–16.6-fold for gallic acid, 1.1–3.5-fold for protocatechuic acid, 1.4–37.0-fold for chlorogenic acid, 2.0–64.0-fold for albiflorin, 2.1–8.7-fold for paeoniflorin, 1.1-fold for ferulic acid, 5.9–57.1-fold for liquiritin, 1.1–7.8-fold for benzoic acid, 5.0–224.5-fold for nodakenin, 5.5–624.9-fold for hesperidin, 1.6–8.5-fold for naringin, 2.4–73.0-fold for neohesperidin, 3.5–13.8-fold for ononin, 3.8–71.8-fold for oxypeucedanin hydrate, 3.7–21.3-fold for cinnamic acid, 5.0–124.0-fold for byakangelicin, 1.5-fold for cinnamaldehyde, 3.8–112.6-fold for benzoylpaeoniflorin, and 3.1–8.6-fold for glycyrrhizin (Table 5).

This result implies that the water extract and commercial granules of OJS were not chemically equivalent because of the variation in the content of the marker compounds.

### 3.4. Evaluation of Correlation between the OJS Samples Using Chemometric Analysis

**Figure 3:**
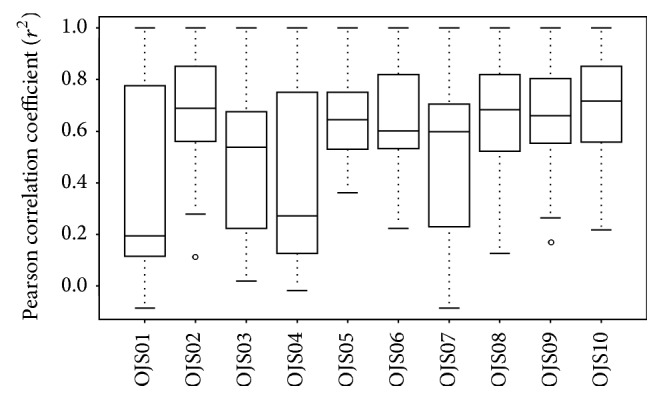
Pearson correlation coefficient (*r* ^2^) between the OJS samples. OJS01, Ojeok-san water extract from the laboratory; OJS02–OJS10, Ojeok-san granules from Korean manufacturers.

Similarities between the OJS samples were assessed using the Pearson correlation coefficient (*r* ^2^), which is a measurement of the distance between two samples and shows the degree of their relationship: a stronger correlation is observed when *r* ^2^ is closer to a value of 1 [22]. The average value of *r* ^2^ for OJS01 was the lowest, followed by OJS04, while the values of the other OJS samples were in the range 0.5 < *r* ^2^ < 0.8 (Figure 3). This means that OJS01 and OJS04 were weakly correlated with the other OJS granules, which showed a mild correlation between samples [23].

Clustering is a partitioning process of objects set into disjoint clusters: objects in the same cluster are similar, while objects belonging to different clusters differ considerably according to their attributes [24], to which PCA and HCA can then be applied.

**Figure 4:**
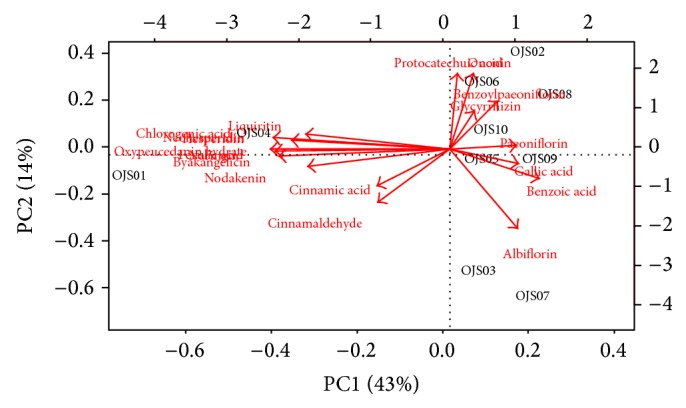
Biplot of the principal components (PC1 versus PC2) of the variables (the contents of the 19 marker compounds) with the objectives (OJS samples). The effect of the marker compounds on the distribution of OJS samples is shown by the red-colored arrows. PC1 and PC2 contributed to 43% and 14% of total variance, respectively. OJS01, Ojeok-san water extract from the laboratory; OJS02–OJS10, Ojeok-san granules from Korean manufacturers.

The 10 OJS samples were distributed on a PCA plot using their PC1 and PC2 scores, as these had higher eigenvalues and, thus, contained the chemically relevant variance [25]. OJS01 and OJS04 had a negative PC1 score, while the other samples had a positive PC1 score, and these were further divided by their PC2 score. The laboratory OJS water extract was differentiated from the commercial OJS granules, except for OJS04, by its PC1 score, which was the most influential factor for clustering the samples. Moreover, the distribution of the commercial OJS samples, especially OJS03 and OJS07, was not located close to each other but spread wide by their PC2 score. Therefore, this was a lower influential factor on the clustering samples after the PC1 score. The marker compounds contributing to the distribution of OJS samples were mainly cinnamic acid, cinnamaldehyde, albiflorin, and benzoylpaeoniflorin, which are denoted by the red-colored arrows in the PCA plot in Figure 4.

**Figure 5:**
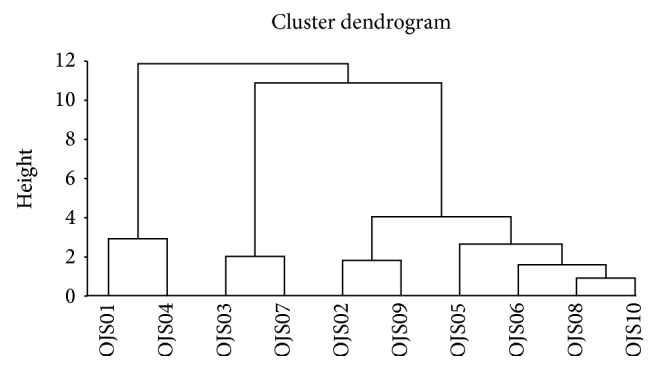
Dendrogram of the hierarchical clustering of OJS samples. OJS01, Ojeok-san water extract from the laboratory; OJS02–OJS10, Ojeok-san granules from Korean manufacturers.

HCA is a method used to measure the distance between objects and find the underlying structure. It uses an iterative procedure that either associates or dissociates a group object by object to classify objects [26]. New clusters are produced by measuring the smallest increase in the sum of the squared within-cluster distances between all the possible clusters, and these are represented by dendrograms [27]. The 10 OJS samples were classified using Ward's method employing the Euclidean distance as a measurement for the HCA. OJS01 showed an exclusively close correlation with OJS04 and formed a separate cluster from the other commercial samples. These were segregated at a height around a value of 11. Under a height value around 5, the commercial OJS samples were further divided into two groups, namely OJS03 and OJS07, and OJS02, OJS05, OJS06, and OJS08–OJS10, which is similar to the results from the PCA analysis (Figure 5).

Taking the results of the quantification and chemometric analyses together, the OJS water extract (OJS01) produced in the laboratory showed little correlation with the commercially manufactured OJS granules from a chemical perspective. This result demonstrates that the low correlation between the OJS samples, particularly the laboratory-produced water extract and the commercial granules, can presumably be ascribed to the different ratios of the compositional herbal medicines, herbal resources, or extraction procedures of the OJS preparations between different pharmaceutical companies.

Therefore, verification of the herbal resources, using an identical combination ratio, or using a valid extraction process, is required to produce chemically equalized OJS preparations that can guarantee an equivalent therapeutic efficacy.

## 4. Conclusions

The analytical method developed using an HPLC-PDA with a reversed-phase C_18_ column was precise, accurate, and reliable and was successfully applied to the simultaneous determination and quantification of 19 marker compounds for the quality assessment of OJS samples. The content of the marker compounds varied between the OJS samples. Moreover, a laboratory-produced OJS water extract was not closely related to the commercial OJS granules, which also showed a wide distribution in the results of chemometric analyses, such as the Pearson correlation coefficient, PCA, and HCA. Our results suggest that HPLC–PDA combined with chemometric analysis can be a useful strategy for the quality evaluation of OJS samples from different origins. It is necessary to produce chemically equalized OJS preparations for better quality samples.

## Figures and Tables

**Table 1:** Compositional ratio of herbal medicine consisting of Ojeok-san (OJS) samples.

Herbal medicine	OJS01^a^	OJS02^b^	OJS03	OJS04	OJS05	OJS06	OJS07	OJS08	OJS09	OJS10
Atractylodis rhizoma	0.133	0.212	0.215	—	0.212	0.443	0.212	0.532	0.214	0.443
Ephedrae herba	0.067	0.045	0.046	—	0.045	0.223	0.045	0.268	0.045	0.223
Citri Unshius pericarpium	0.067	0.091	0.090	—	0.091	0.223	0.091	0.268	0.090	0.223
Magnoliae cortex	0.053	0.018	0.018	—	0.018	0.223	0.018	0.268	0.018	0.223
Platycodonis radix	0.053	0.095	0.097	—	0.095	0.223	0.095	0.268	0.097	0.223
Aurantii Fructus Immaturus	0.053	0.069	0.070	—	0.069	—	0.069	—	0.070	—
Angelicae gigantis radix	0.053	0.082	0.084	—	0.082	0.223	0.082	0.268	0.083	0.223
Zingiberis rhizoma	0.053	0.049	0.050	—	0.049	0.223	0.049	0.268	0.050	0.223
Paeoniae radix	0.053	0.060	0.061	—	0.060	0.223	0.060	0.268	0.061	0.223
*Poria* sclerotium	0.053	0.004	0.004	—	0.004	0.223	0.004	0.268	0.004	0.223
Cnidii rhizoma	0.047	0.069	0.067	—	0.069	0.223	0.069	0.268	0.066	0.223
Angelicaedahuricae radix	0.047	0.071	0.069	—	0.071	0.223	0.071	0.268	0.069	0.223
Pinelliae tuber	0.047	0.051	0.050	—	0.051	0.223	0.051	0.268	0.050	0.223
Cinnamomi cortex	0.047	0.011	0.011	—	0.011	—	0.011	0.000	0.011	—
Glycyrrhizaeradix et rhizoma	0.040	0.045	0.046	—	0.045	0.223	0.045	0.268	0.045	0.223
Zingiberis rhizoma crudus	0.067	0.027	0.028	—	0.027	—	0.027	—	0.027	—
Ponciri Fructus Immaturus	—	—	—	—	—	0.223	—	0.268	—	0.223
Zizyphi Fructus	—	—	—	—	—	0.223	—	0.268	—	0.223
Cinnamomi ramulus	—	—	—	—	—	0.223	—	0.268	—	0.223
Cyperi rhizoma	—	—	—	—	—	—	—	0.160	—	0.133
Allii fistulosi bulbus	0.067	—	—	—	—	—	—	—	—	—

Single dose	1	1	1	—	1	1	1	1	1	1

^a^OJS01, Ojeok-san water extract from the laboratory and ^b^OJS02–OJS10 = Ojeok-san granules from Korean manufacturers.

**Table 2:** Regression, correlation coefficient (*r* ^2^), LOD, and LOQ of the marker compounds of OJS.

Compound	UV wavelength	Regression equation		Linear range (*μ*g/mL)	*r* ^2^	LOD (*μ*g/mL)	LOQ (*μ*g/mL)
		Slope	Intercept				
Gallic acid	270 nm	39,349	4,315	0.63–5.00	0.9995	0.015	0.047
Protocatechuic acid	260 nm	42,285	2,576	0.31–5.00	0.9994	0.014	0.043
Chlorogenic acid	325 nm	32,676	9,500	1.56–25.00	0.9994	0.019	0.056
Albiflorin	230 nm	10,703	2,218	1.56–25.00	0.9998	0.057	0.172
Paeoniflorin	230 nm	15,956	−377	4.69–75.00	1.0000	0.038	0.115
Ferulic acid	325 nm	44,533	17,050	1.56–25.00	0.9993	0.014	0.041
Liquiritin	275 nm	24,585	12,410	4.69–75.00	0.9999	0.025	0.075
Benzoic acid	230 nm	38,560	11,910	1.56–25.00	0.9998	0.016	0.048
Nodakenin	335 nm	34,254	17,529	4.69–75.00	0.9999	0.018	0.054
Hesperidin	280 nm	18,406	25,320	10.94–175.00	0.9999	0.033	0.100
Naringin	280 nm	15,468	20,566	10.94–175.00	0.9999	0.039	0.119
Neohesperidin	280 nm	25,094	24,132	7.81–125.00	0.9999	0.024	0.073
Ononin	250 nm	58,807	3,317	0.31–5.00	0.9994	0.010	0.031
Oxypeucedanin hydrate	310 nm	16,087	2,865	1.56–25.00	0.9993	0.038	0.114
Cinnamic acid	275 nm	93,234	10,584	0.63–10.00	0.9998	0.007	0.020
Byakangelicin	270 nm	23,738	5,392	1.56–25.00	0.9994	0.026	0.077
Cinnamaldehyde	290 nm	156,619	8,846	0.33–5.25	0.9996	0.004	0.012
Benzoylpaeoniflorin	230 nm	28,272	274	0.16–2.50	0.9997	0.021	0.065
Glycyrrhizin	250 nm	6,765	1,864	1.56–25.00	0.9993	0.090	0.272

**Table 3:** Intra- and interday precision of the marker compounds of OJS.

Compound	Spiked concentration (*μ*g/mL)	Intraday (*n* = 3)			Interday (*n* = 3)		
		Detected concentration (*μ*g/mL)	RSD^a^ (%)	Accuracy (%)	Detected concentration (*μ*g/mL)	RSD (%)	Accuracy (%)
Gallic acid	1.00	1.00	1.68	100.41	1.00	1.68	100.41
	2.00	2.00	0.37	100.18	2.00	0.48	100.12
Protocatechuic acid	1.00	1.02	0.89	101.59	1.00	1.79	100.11
	2.00	2.00	0.45	99.97	2.01	0.43	100.31
Chlorogenic acid	2.00	1.96	0.27	98.04	1.96	0.38	97.98
	4.00	4.02	0.06	100.49	4.02	0.09	100.51
Albiflorin	2.00	1.99	1.09	99.56	1.97	2.57	98.61
	4.00	4.00	0.27	100.11	4.02	0.45	100.52
Paeoniflorin	10.00	10.22	1.36	102.20	10.22	1.37	102.20
	20.00	19.89	0.35	99.45	19.89	0.35	99.45
Ferulic acid	2.00	2.01	0.56	100.53	1.99	1.10	99.62
	4.00	4.00	0.26	100.09	4.00	0.27	100.10
Liquiritin	5.00	5.22	1.13	104.46	5.24	1.50	104.80
	10.00	9.89	0.30	98.88	9.88	0.40	98.80
Benzoic acid	3.00	2.88	0.67	95.97	2.87	1.22	95.67
	6.00	6.06	0.16	101.01	6.06	0.29	101.08
Nodakenin	5.00	5.01	0.42	100.12	5.03	0.28	100.51
	10.00	10.00	0.11	99.97	9.99	0.16	99.93
Hesperidin	20.00	20.59	0.39	102.97	20.60	0.41	102.98
	40.00	39.70	0.10	99.26	39.70	0.11	99.25
Naringin	20.00	20.83	0.30	104.15	20.83	0.31	104.15
	40.00	39.58	0.08	98.96	39.58	0.08	98.96
Neohesperidin	15.00	14.11	0.15	94.07	14.10	0.23	94.02
	30.00	30.44	0.03	101.48	30.46	0.02	101.53
Ononin	1.00	0.98	0.26	97.60	0.98	0.24	97.60
	2.00	2.01	0.06	100.60	2.01	0.06	100.60
Oxypeucedanin hydrate	1.00	0.98	1.99	97.91	0.97	2.65	97.35
	2.00	2.01	0.49	100.52	2.01	0.64	100.66
Cinnamic acid	1.00	0.99	0.09	99.26	0.99	0.50	99.02
	2.00	2.00	0.02	100.19	2.00	0.12	100.24
Byakangelicin	1.00	0.99	1.53	99.25	0.99	1.87	99.01
	2.00	2.01	0.22	100.49	2.01	0.36	100.59
Cinnamaldehyde	1.05	1.02	0.63	97.43	1.03	0.93	97.64
	2.10	2.11	0.31	100.27	2.11	0.34	100.29
Benzoylpaeoniflorin	1.00	1.01	1.53	100.87	1.00	1.29	99.81
	2.00	2.00	0.32	100.05	2.00	0.45	99.97
Glycyrrhizin	3.00	2.93	0.38	97.53	2.92	0.68	97.35
	6.00	6.04	0.09	100.62	6.04	0.16	100.66

^a^RSD (%) = (SD/mean) × 100.

**Table 4:** Recovery of the marker compounds of OJS (*n* = 3).

Compound	Initial concentration (*μ*g/mL)	Spiked concentration (*μ*g/mL)	Detected concentration (*μ*g/mL)	Recovery (%)	RSD (%)^a^
Gallic acid	1.90	1.00	2.88	98.05	2.28
		2.00	3.82	96.15	3.65
Protocatechuic acid	0.39	1.00	1.41	102.22	1.75
		2.00	2.47	104.16	0.88
Chlorogenic acid	6.52	2.00	8.37	92.74	1.21
		4.00	10.36	95.89	1.08
Albiflorin	3.62	2.00	5.57	97.19	1.41
		4.00	7.61	99.59	2.80
Paeoniflorin	15.89	10.00	25.84	99.54	1.86
		20.00	34.91	95.12	0.78
Ferulic acid	3.75	2.00	5.65	95.33	1.09
		4.00	7.53	94.58	0.24
Liquiritin	16.01	5.00	20.98	99.43	1.74
		10.00	25.16	91.55	0.43
Benzoic acid	6.72	3.00	9.59	95.74	1.91
		6.00	12.84	101.90	1.70
Nodakenin	8.98	5.00	13.74	95.28	1.03
		10.00	18.40	94.27	0.77
Hesperidin	61.70	20.00	81.66	99.77	0.28
		40.00	99.80	95.26	0.56
Naringin	62.30	20.00	82.34	100.19	0.72
		40.00	99.63	93.32	0.38
Neohesperidin	36.31	15.00	50.24	92.90	0.18
		30.00	66.90	101.97	0.07
Ononin	0.56	1.00	1.50	94.75	0.05
		2.00	2.52	98.29	0.35
Oxypeucedanin hydrate	7.18	1.00	8.11	93.19	2.25
		2.00	9.14	97.97	2.43
Cinnamic acid	0.94	1.00	1.92	97.97	0.84
		2.00	2.93	99.43	0.25
Byakangelicin	4.95	1.00	5.94	99.43	1.40
		2.00	6.96	100.58	1.67
Cinnamaldehyde	1.66	1.05	2.63	92.64	1.97
		2.10	3.66	95.06	1.17
Benzoylpaeoniflorin	0.38	1.00	1.39	100.55	1.19
		2.00	2.40	100.85	1.95
Glycyrrhizin	23.08	3.00	26.00	97.50	0.47
		6.00	29.19	101.91	0.83

^a^RSD (%) = (SD/mean) × 100.

**Table 5:** The average content of the marker compounds in OJS samples (*n* = 3).

Compound	Content (mg/g)									
	OJS01^a^	OJS02^b^	OJS03	OJS04	OJS05	OJS06	OJS07	OJS08	OJS09	OJS10
Gallic acid	0.096 ± 0.001	0.609 ± 0.023	0.978 ± 0.054	0.531 ± 0.005	0.273 ± 0.003	0.372 ± 0.004	0.371 ± 0.013	0.372 ± 0.023	1.592 ± 0.068	0.379 ± 0.006
Protocatechuic acid	0.019 ± 0.000	0.063 ± 0.001	ND^c^	0.032 ± 0.000	0.022 ± 0.002	0.019 ± 0.000	ND	0.018 ± 0.001	0.035 ± 0.001	0.036 ± 0.002
Chlorogenic acid	0.326 ± 0.002	0.009 ± 0.002	ND	0.333 ± 0.014	0.013 ± 0.000	0.082 ± 0.006	ND	0.028 ± 0.001	0.023 ± 0.002	0.036 ± 0.014
Albiflorin	0.184 ± 0.008	1.635 ± 0.020	5.950 ± 0.066	0.249 ± 0.010	0.243 ± 0.006	0.093 ± 0.011	5.060 ± 0.071	0.938 ± 0.017	1.072 ± 0.015	0.701 ± 0.040
Paeoniflorin	0.796 ± 0.006	3.363 ± 0.030	2.457 ± 0.046	2.294 ± 0.030	0.385 ± 0.017	1.529 ± 0.034	2.190 ± 0.048	1.679 ± 0.005	2.522 ± 0.052	1.539 ± 0.010
Ferulic acid	0.187 ± 0.000	ND	ND	0.172 ± 0.004	ND	ND	ND	ND	ND	ND
Liquiritin	0.800 ± 0.003	0.325 ± 0.008	0.259 ± 0.011	0.237 ± 0.009	0.083 ± 0.000	0.214 ± 0.005	0.014 ± 0.002	0.247 ± 0.002	0.236 ± 0.003	0.150 ± 0.003
Benzoic acid	0.337 ± 0.003	0.950 ± 0.012	0.449 ± 0.003	0.371 ± 0.007	0.244 ± 0.001	0.275 ± 0.001	1.902 ± 0.103	0.998 ± 0.005	1.612 ± 0.005	0.476 ± 0.003
Nodakenin	0.449 ± 0.002	ND	0.037 ± 0.000	0.002 ± 0.001	0.010 ± 0.000	ND	0.057 ± 0.001	0.045 ± 0.000	0.030 ± 0.001	ND
Hesperidin	3.086 ± 0.003	0.437 ± 0.013	0.348 ± 0.004	4.999 ± 0.010	0.376 ± 0.001	0.875 ± 0.006	ND	0.044 ± 0.001	0.214 ± 0.002	0.008 ± 0.002
Naringin	3.115 ± 0.002	0.578 ± 0.017	1.084 ± 0.007	2.983 ± 0.001	0.631 ± 0.001	0.783 ± 0.002	0.366 ± 0.004	0.580 ± 0.001	1.276 ± 0.004	0.750 ± 0.003
Neohesperidin	1.816 ± 0.001	0.277 ± 0.001	0.198 ± 0.001	3.139 ± 0.003	0.235 ± 0.000	0.592 ± 0.001	ND	ND	0.105 ± 0.000	0.043 ± 0.001
Ononin	0.028 ± 0.000	0.038 ± 0.000	0.022 ± 0.000	0.018 ± 0.000	0.014 ± 0.000	0.049 ± 0.000	0.004 ± 0.000	0.055 ± 0.000	0.033 ± 0.000	0.028 ± 0.000
Oxypeucedanin hydrate	0.359 ± 0.001	0.021 ± 0.001	0.052 ± 0.000	0.165 ± 0.000	0.028 ± 0.001	0.057 ± 0.000	0.005 ± 0.000	0.028 ± 0.001	0.019 ± 0.000	0.080 ± 0.001
Cinnamic acid	0.047 ± 0.000	0.033 ± 0.000	0.022 ± 0.000	0.055 ± 0.001	0.003 ± 0.000	0.011 ± 0.001	0.064 ± 0.002	0.013 ± 0.001	0.023 ± 0.000	0.041 ± 0.000
Byakangelicin	0.248 ± 0.002	0.013 ± 0.000	0.046 ± 0.000	0.071 ± 0.001	0.010 ± 0.001	0.030 ± 0.000	0.002 ± 0.000	0.016 ± 0.000	0.015 ± 0.000	0.026 ± 0.000
Cinnamaldehyde	0.083 ± 0.001	ND	0.122 ± 0.002	ND	ND	ND	ND	ND	ND	ND
Benzoylpaeoniflorin	0.019 ± 0.001	0.563 ± 0.006	0.049 ± 0.001	0.058 ± 0.004	0.005 ± 0.000	0.049 ± 0.001	0.048 ± 0.002	0.107 ± 0.001	0.052 ± 0.000	0.038 ± 0.002
Glycyrrhizin	1.155 ± 0.005	1.101 ± 0.032	0.959 ± 0.034	0.762 ± 0.044	0.248 ± 0.023	2.025 ± 0.015	1.179 ± 0.038	2.124 ± 0.028	0.790 ± 0.049	1.445 ± 0.037

The average content is represented as mean ± SD.

^a^OJS01, Ojeok-san water extract from the laboratory, ^b^OJS02–OJS10 = Ojeok-san granules from Korean manufacturers, and ^c^ND, not detected.
